# The impact of the COVID-19 pandemic on Industrial Organisational Psychologists in South Africa: Imagining new professional roles

**DOI:** 10.4102/sajip.v47i0.1876

**Published:** 2021-11-18

**Authors:** Willie T. Chinyamurindi, Anthony K. Masha, Ntombekhaya Tshabalala

**Affiliations:** 1 Department of Business Management, Faculty of Management and Commerce, University of Fort Hare, East London, South Africa; 2 Department of Management, Walter Sisulu University, Queenstown, South Africa

**Keywords:** COVID-19, industrial organisational psychology, industrial organisational psychologists, individuals, organisations, roles

## Abstract

**Orientation:**

The coronavirus disease 2019 (COVID-19) pandemic has affected the world of work. An understanding is needed of this impact and the positioning of roles of professional psychologists in response to adjusting to the new normal.

**Research purpose:**

This study investigated the challenges faced by Industrial Organisational (IO) Psychologists in view of the COVID-19 pandemic. Furthermore, the study aimed at ascertaining the professional roles that emanate from such challenges.

**Motivation of the study:**

As a result of the COVID-19 pandemic, calls exist within the literature for nuanced disciplinary studies that explore the impact of the pandemic. One such discipline is that of Industrial Psychology (IP), a discipline deemed important not only for the development of individuals but also for organisations.

**Research approach, design and method:**

A qualitative research approach utilising semi-structured interviews was conducted with 25 IO Psychologists. Thematic analysis was utilised to analyse the collected data using the suggestions by Braun and Clarke.

**Main findings:**

Two main findings emerged from the study informed by the thematic analysis conducted. Firstly, the IO Psychologists expressed challenges of a direct nature affecting their practice and individual well-being. Secondly, the participating IO Psychologists suggested resultant professional roles that emerged from the impact of the COVID-19 pandemic. These included (1) prioritisation of personal physical and mental health, (2) more technological skills and acumen needed to adjust to the challenges posed by the pandemic, (3) promotion of continued professional learning and (4) the necessity for support networks amongst practitioners.

**Practical/managerial implications:**

Implications are drawn for the practitioners and individuals working within the IO Psychology context. These extend at assisting the practitioners within the presented challenges.

**Contribution/value-add:**

Through the findings, suggestions to inform IO Psychology as a practice are made. Furthermore, roles in assisting IO Psychologists to adjust to the new normal are suggested. The study becomes one of the first within these disciplines to charter suggestions for this important practice.

## Introduction

In 2019, the world witnessed the devastating impact of the novel coronavirus disease 2019 (COVID-19). COVID-19 is the novel coronavirus that goes with the name severe respiratory syndrome coronavirus-2 (SARS-COV-2) (Sansa, 2020). The virus was first identified in Wuhan City in China at the end of 2019 and is associated by scientists with a disease referred to as COVID-19 (Sansa, 2020). Researchers at Imperial College in London estimated the global impact of the COVID-19 virus to range between 20 million deaths in 2020, with effective non-pharmaceutical interventions in place and 40 million deaths, without such interventions (Walker, Whittaker, & Watson, 2020).

As a result of the COVID-19 virus, there was a drastic change of life (World Health Organization, 2020), leading to the COVID-19 pandemic raising some glaring issues. Firstly, the observed impact was not only on economic growth but also on global financial markets (Baur, 2020). Some predictions estimated that the global economy could witness its slowest ever growth rate since 2009 (Organisation for Economic Co-operation and Development, 2020). On the business front, expectations were that businesses would be affected by the pandemic, including the management of people (Bapuji et al., 2020). There is acknowledgement of the need to adjust to the new normal and a move towards some form of normality given the difficulty posed by the COVID-19 pandemic (Handfield, Graham & Burns 2020; Trautrims, Schleper, Cakir & Gold, 2020).

It is also acknowledged that the COVID-19 pandemic has a compounding effect, especially on the vulnerable in society (Raju & Van Niekerk, 2020), inclusive of their area of habitation (Raju & Ayeb-Karlsson, 2020). Such a state of affairs exists as a threat to individuals’ health and well-being (Beckman, Mechanic, Shah, & Figueroa, 2021). In countries such as South Africa, the COVID-19 pandemic is believed to exacerbate the already existing challenges of poverty (Van der Merwe, 2020). The COVID-19 pandemic has impacted even the work of the non-governmental sector with devastating consequences (Ned, McKinney, McKinney, & Swartz, 2020).

Given the challenge of the COVID-19 pandemic, some calls to address COVID-19 related challenges exist within the literature. Firstly, there is a need to be expansive whilst understanding the impact of the COVID-19 pandemic and explore the support provided. The focus then is on understanding micro issues and linkages with macro issues stemming from the impact of the COVID-19 pandemic (Cho, 2020). Emerging empirical focus within South Africa has been centred on understanding the impact of the COVID-19 pandemic at a wider societal level, including interventions for support (De Klerk, Joubert, & Mosca, 2021). This places importance on understanding the impact of the COVID-19 pandemic and the targeted interventions from an organisational and institutional unit of analysis (Magezi & Manzanga, 2020). This has seen studies emerge from an organisational lens and relying mostly on managers and employees as informants (Atiku, Jeremiah, & Boateng, 2020). Secondly, calls exist for more disciplinary contributions to understanding the COVID-19 pandemic. This is observed within the psychology discipline concerned with understanding ‘human behaviour’, warranting further inquiry in how the pandemic as a challenge can be addressed (Pillay & Barnes, 2020, p. 148).

Given the evolving nature of developments around the COVID-19 pandemic, the voices of experts within the field and disciplines of Industrial Organisation (IO) Psychology can be useful in enhancing understanding of the unfolding complexity. An IO Psychologist is viewed through the South African Health Professions Act as an individual who assists adults in adjusting to work-related issues by applying psychology principles (South African Department of Health, 2012). IO Psychologists are deemed an important conduit in assisting employees with counselling related to personal and work problems (De jager-van Straaten, Jorgensen, Hill, & Nel, 2016). With the ambivalence that exists around the current business environment given the COVID-19 pandemic (McKinsey 2020), IO Psychologists’ role may be deemed critical in such a situation.

Practitioners within the IO Psychology discipline become important both for the individual and for the organisational growth (Bergh, 2012). Furthermore, IO Psychologists seek to maintain a fit between an employee and their workplace (Schreuder & Coetzee, 2010). IO Psychology’s strength is not their knowledge of theory but its application to real-life scenarios (Cilliers & Flotman, 2016). Given this important role of IO Psychologists, there is also a need to understand such practitioners’ views, especially regarding the changing nature of work and the impact of the COVID-19 pandemic. As evident in emerging international work, the benefit here is the potential to understand the impact of the COVID-19 pandemic and to link this with already existing issues and challenges on the ground (Blustein et al., 2020). This becomes important, especially within a South African context, as IO Psychology has been framed to have a more community development function (Van Zyl, Nel, Stander, & Rothmann, 2016).

## Research purpose and objectives

The scope of this research is within the field of IO Psychology. The views and opinions of IO Psychologists on how the COVID-19 pandemic will affect their discipline can be a useful precursor to interventions that assist such practitioners. Calls exist within the literature for sector influenced responses to the challenges of the COVID-19 pandemic in response to discipline and community needs (Hudecheck, Siren, Grichnik, & Wincent, 2020). The research thus sought to understand the framing of the COVID-19 pandemic to IO Psychology as a practice and explore the role of IO Psychologists as a result of all this. The following research question was set: *How do IO Psychologists frame the COVID-19 pandemic as part of their personal and professional experience and what envisaged role do they play as part of this framing?*

## Literature review

### Theoretical lens

The study has borrowed from two main theoretical considerations. Firstly, the human capital theory: At the core of the human capital theory is the emphasis on human resources existing as a competitive advantage source (Bag & Gupta, 2019). This places importance on the existence of such resources and also on their management (Hofmann & Rüsch, 2017). In response to challenges, organisations can use internal or external capabilities for their advantage (Barreto, Amaral, & Pereira, 2017). This can include the use of technology, changes to organisational structure and work plans to respond to the challenges experienced (Telukdarie, Buhulaiga, Bag, Gupta, & Luo, 2018). This can be a source of competitive advantage (Evans, 2019). A second theoretical consideration is the job demands-resources model (Bakker & Demerouti, 2007) and its emphasis on the individual and the demands and resources needed as a part of the employment experience (Carlson et al., 2017). The demands can exert psychological and physical strain on the individual (Bakker & Demerouti, 2007). Resources then emerge to manage such strain and consist of dimensions of a cognitive, social and psychological nature in promoting employee well-being (Ryff, 2013).

### Empirical literature

The first quarter of 2020 was a hard time for the global community with the outbreak of the COVID-19 virus (Demuyakor, 2020), which forced the World Health Organisation (WHO) to declare it a global pandemic (Adu, Mpu, & Adu, 2020). As an unprecedented phenomenon in history (Yong, 2020), the outbreak of the COVID-19 pandemic saw the largest disruption in history by causing worldwide disruption in literally all spheres of life (UN Policy Brief: Education during COVID-19 and beyond, 2020). This is acknowledged by Hlatshwayo (2020) as a major challenge, especially in contemporary society. Therefore, to understand the impact of the COVID-19 pandemic on aspects related to work, there is a need to acknowledge the already precarious situation prevailing pre-COVID-19.

#### Impact of the COVID-19 pandemic

The COVID-19 pandemic has affected the personal and professional lives of individuals. The pandemic has led to changes that have impacted not only individuals but also the industries they operate in (Adu et al., 2020). One such impact has been remote working with technology occupying a huge role (Abdulamir & Hafidh, 2020; Di Pietro, Biagi, Costa, Karpiński, & Mazza, 2020; Raheem & Khan, 2020). Regrettably, as a result of the pandemic, this in turn has led to structural changes to work and to job losses (Bawa, 2020; Demuyakor, 2020; Mhlanga & Moloi, 2020). Such a situation affects the individual, especially mentally (Di Pietro et al., 2020).

A general assumption, being supported by evidence, is that the pandemic impacts decent work conditions. This includes observed changes in work conditions and notably a reduction in the hours of work (International Labor Organisation, 2020b) and a possible increase in the unemployment rate (International Labor Organisation, 2020c). Others believe the impact of the COVID-19 pandemic to be negative and with some possible positives to emerge in assisting affected individuals (Akkermans, Richardson & Kraimer, 2020). One such belief is the need to revisit organisational policies to be in line with new work arrangements (Russell, 2019). This behaviour is observed as common, especially when organisations are faced with a period of uncertainty (Del Giudice et al., 2017; Reimers & Schleicher, 2020).

Given these challenges posed by the COVID-19 pandemic affecting the nature of work, the situation appears dire for those who are still in employment. The situation has resulted in job security concerns and heightened anxiety amongst those already employed (International Labor Organization, 2020a; Kelly, 2020). The situation is made worse especially for those who were unemployed, given their already existing challenges (Hooley, Sultana, & Thomsen, 2000). In essence, the entire labour market system can be considered as experiencing a career shock as a result of the COVID-19 pandemic (Cox, 2020a, 2020b). Given such a situation, not only is an individual response needed but one that is also cognisant of the organisation (Van Hoek, 2020).

#### Effect of the COVID-19 pandemic

Given the presented impact, there is a need to ascertain the role that IO Psychology can play (Rudolph & Zacher, 2020) to proffer a more nuanced position around experiences related to the impact of the COVID-19 pandemic. This argument is heightened in the work of Guan, Deng and Zhou (2020) who argued for the need of a response to COVID-19 to be culturally sensitive to local conditions and issues. Furthermore, there is a continued need to understand how a range of organisations respond to COVID-19 and its impact on the firm’s operation and performance (Rapaccini, Saccani, Kowalkowski, Paiola, & Adrodegari, 2020).

A prediction exists that more individuals will begin to develop extra skills to remain employable (Jerman, Pejić Bach, & Aleksić, 2020), given the possibility of job losses. This individual effort has the potential to enhance organisations in the long run. Skills that will be needed in a post-COVID-19 world centre around the acquisition of digital skills. This necessitates individuals being cognisant in the usage of technologies that enhance connectivity, such as telecommuting, virtual teams and cloud computing (Li, 2016).

In countries such as Namibia, flexibility and technology have helped employers and employees respond to the COVID-19 pandemic (Atiku et al., 2020). Some caution against the need for technology preparedness, especially given the proliferation of technology usage (Naidoo, 2020). This has led to organisational and individual preparedness in acquiring digital literacy skills (Chinyamurindi, 2020a). A starting point may be to manage prevailing negative thoughts and stereotyping that involve the role of technology within the confines of work (Chinyamurindi, 2020b), including attitudes towards technology, especially during the pandemic (Jere, 2020).

Within a post-COVID world, as in the current situation, there will be a continued emphasis on promoting aspects related to health and safety including general well-being. This becomes important keeping in mind the concept of inclusion especially as individuals find their way in a difficult world. There should thus be emphasis on the importance of continued efforts that promote health within the workplace (Ipsen, Kirchner, & Hansen, 2020). Given the lessons learned during the pandemic, a post-COVID-19 world is predicted to place more awareness on issues that promote employee health and safety (O’Connor et al., 2020).

As a response to the COVID-19 pandemic, there is a need for IO Psychologists and researchers to be responsive to the challenges and opportunities posed by the COVID-19 pandemic in a proactive manner (Rudolph et al., 2020). Challenges such as mental health issues will affect individuals during and post-COVID-19 era (Akhter-Khan & Wai, 2020). In Singaporean organisations, calls have been made for effective and efficient organisational support structures that should be in place to assist individuals as they navigate through the challenges posed by the COVID-19 pandemic (Lee, Goy, Sng, & Lew, 2020). Efforts and experiences of managing the COVID-19 pandemic can be instrumental in preparing for future pandemics and disasters that may result (Auer, 2021).

### Research method

An interpretivist research philosophy hinging on the qualitative research method was adopted for this study. There is an acknowledgement of the merit of such a philosophy and research approach, especially in understanding complexity that may accompany the human experience (Chinyamurindi, 2020c) and enable the social researcher to appreciate the subjective meaning of social action (Bryman et al., 2018; Delport, Fouché & Schurink, 2018).

### Sampling and research participants

A non-probability sampling approach was used, relying on a purposive sampling technique because the participants were deemed as ‘information-rich’ and illuminative, that is, they would offer useful manifestations of the impact of the COVID-19 pandemic on Industrial Organisational (IO) Psychologists in South Africa (Cohen, Manion, & Morrison, 2018; Rule & John, 2017). The aim was to narrow focus only on those characteristics needed in the sample (Mooi, Sarstedt, & Mooi-Reci, 2018; Morgan & Sklar, 2018; Nardi, 2018), that is, experts within the IO Psychology field. The key informants were identified through known contacts and the following professional bodies: (1) South African Board for People Practices, (2) the Psychological Society of South Africa, (3) South African Career Development Association and (4) the Institute for People Management. A total of 25 IO Psychologists, of which 11 white people, 7 black people, 5 coloured and 2 Indians, took part in the research, with data being collected over a 6-month period (see Table 1 for demographic characteristics of participants).

**TABLE 1:** Demographic characteristics of participants.

Participant	Gender (race)	Years of experience as an IOP	Location
1	Female (W)	7	Johannesburg
2	Male (W)	6	Cape Town
3	Female (C)	10	Johannesburg
4	Female (W)	15	Port Elizabeth
5	Male (B)	13	Pretoria
6	Male (C)	11	Cape Town
7	Male (B)	4	Port Elizabeth
8	Female (W)	4	Johannesburg
9	Female (W)	9	Pretoria
10	Female (C)	14	Cape Town
11	Female (W)	8	Johannesburg
12	Female (W)	10	Pretoria
13	Female (I)	16	Pretoria
14	Male (B)	9	Johannesburg
15	Female (B)	8	Pretoria
16	Female (C)	10	Bloemfontein
17	Male (B)	11	Port Elizabeth
18	Male (W)	15	Pretoria
19	Male (W)	15	Johannesburg
20	Male (W)	11	Durban
21	Male (C)	9	Johannesburg
22	Male (B)	10	Pretoria
23	Female (I)	15	Durban
24	Male (B)	4	Pretoria
25	Male (W)	7	Johannesburg

W, white person; B, black person; C, coloured person; I, Indian person; IOP, Industrial Organisational Psychologist.

The following inclusion and exclusion criteria were used to select participants: (1) a participant had to be a registered IO Psychologist in good standing with the Health Professions Council of South Africa and (2) a participant had to be classified as an expert in any of the IO Psychology discipline areas. To be considered an expert, participants had to have at least 4 years of work experience within their discipline area. The key informants further used these criteria in the recruiting process. This ensured consistency in the information gathered from the participants’ experiences. The participants’ demographic characteristics are illustrated in Table 1.

### Data collection and data recording

The guiding aims and research question made it possible to use a qualitative research approach, especially given the complexity that has been created by the COVID-19 pandemic. In achieving all this, semi-structured interviews were utilised with IO Psychologists in practice operating in South Africa. A total of 25 interviews were conducted over the Zoom platform with the IO Psychologists in practice. The interviews were video-recorded with permission. Each interview lasted between 30 and 50 min.

### Strategies to ensure data integrity

There is a need to ensure data quality and reporting. The researcher paid attention to many strategies. Firstly, in terms of credibility, all interviews were conducted and recorded over the Zoom platform. This allowed for a platform for interviewing whilst respecting social distancing rules of COVID-19 and enabling recording (done with permission) of each interview. Secondly, after the data had been collected through the video interviews, transcriptions were made and sent back to the participants to correct anything if the need existed. Thirdly, data were collected over 6 months, and this allowed for data collection work, inclusive of transcriptions, to be done without pressure. The 6-month period also ensured that we managed to capture IO Psychologist experiences at the onset, as well as a few months during the course of the pandemic.

### Data analysis

An interpretivist approach was adopted for data analysis in this study. A qualitative thematic analysis approach was utilised. Over the 6 months of data collection, the interviews were transcribed soon after each interview. The transcripts were then entered into the QSR NVivo 9 data analysis and management software (Reuben & Bobat, 2014). The software was used to code each of the transcripts’ paragraphs into themes and sub-themes. The coding was performed using the participants’ own words wherever possible. However, the researcher did not only rely on the software to develop the themes; it was also used as a complementary method to develop themes. Thematic analysis was utilised to analyse the qualitative data by identifying and examining common patterns within the data (Vaismoradi, Turunen & Bondas, 2013). In analysing the data through thematic analysis, the six steps advocated by Braun, Clarke, Hayfield and Terry (2019), as shown in Table 2, were utilised.

**TABLE 2:** Phases of thematic analysis.

Phase	Description
Familiarising yourself with your data	Actively reading and re-reading data to obtain an overall understanding
General initial codes	Noting important aspects of data
Searching for themes	Identify codes and form codes into themes
Reviewing themes	Relating the themes to codes and the entire data set
Defining and naming themes	Producing clear definitions and names for themes
Producing the report	Final analysis of themes relating to the initial research question

*Source*: Braun, V., Clarke, V., Hayfield, N., & Terry, G. (2019). *Thematic analysis*. Singapore: Springer.

### Ethical considerations

The research adhered to ethical guidelines. Firstly, ethical clearance was applied for and granted by the participating institution, the University of Fort Hare Research Ethics Committee where the researchers are based, reference number: CHI001. Secondly, permission was solicited and granted from participants to the study. The researchers assured participants of their confidentiality, anonymity and privacy (Devlin, 2018; Ferreira, 2018; Patten & Newhart, 2018) and emphasised that pseudonyms would be utilised throughout the research. Thirdly, the researchers also adhered to other ethical considerations that included informed consent (Kumar, 2019; Pajo, 2018; Saunders & Lewis, 2018) and the right to withdraw from the research at any point (Neuman, 2014). Finally, after transcribing the interviews, copies were sent back to the participants as a way of verification. Participants were free to add or correct the transcriptions and return them to the researchers.

## Findings

Three main findings emerged from the study informed by the thematic analysis conducted. Firstly, the participating IO Psychologists narrated experiences of personal challenges related to the COVID-19 pandemic. Secondly and related to the professional sphere, the IO Psychologists expressed challenges of a direct nature affecting their practice and individual well-being. Thirdly, the participating IO Psychologists suggested resultant professional roles that emerged from the impact of the COVID-19 pandemic. These included (1) prioritisation of personal physical and mental health, (2) more technological skills and acumen needed to adjust to the challenges posed by the pandemic, (3) promotion of continued professional learning and (4) the necessity for support networks amongst practitioners. These results are presented in the next section.

### Theme 1: Impact of COVID-19 on the Industrial Psychologist as an individual

The participating IO Psychologists expressed the direct impact of the COVID-19 pandemic. A sub-theme that emerged as a direct impact of the COVID-19 pandemic was the individual IO Psychologists being infected by the virus. One participant narrated this experience:

‘I knew based on the symptoms that something was wrong with my system. Being tested was really a no brainer [*yet still important*]. Upon receiving the text message, I prepared myself for 14 days of hell. Quarantine meant no work. It was tough.’ (Participant 17, Male, Port Elizabeth)

Upon testing positive for the virus, another participant also narrated the impact this had on their work. This participant although, having recovered, expressed challenges related to post-recovery:

‘So I went through whole formality leading to recovery. I thought I was fine, but this was not to be. To this day, I have breathing challenges, and I know I may not have COVID, but I am just not the same. Given this, I have to slow down. This means taking breaks during the period even steam myself.’ (Participant 9, Female, Pretoria)

Participants also narrated a great ordeal of mental challenges as a result of the COVID-19 pandemic. One participant expressed the following:

‘There is a great deal of anxiety and uncertainty. We are all scared. We try to be strong and not let our guard down but each day we realise the depth of difficulty that awaits us. So I am scared, but I also realise that I have little or no control on these things.’ (Participant 4, Female, Port Elizabeth)

Another participant expressed concern about the future of their practice, given the challenges posed by the COVID-19 pandemic:

‘Given the turn of events, we have to be constantly planning according to what is going on. The difficulty is that from a national point of view, we appear to be unsure. This uncertainty is unsettling but also questions the future of what I am doing.’ (Participant 18, Male, Pretoria)

### Theme 2: Impact of COVID-19 on the Industrial Organisation Psychologist as a professional

Participants were also quick to mention the impact of the lockdown restrictions and how they affected IO Psychologists’ work. One participant articulated this with a referral to the stages of the lockdown in South Africa:

‘Stages 5 and 4 were the worst. The work we do requires social interaction and these stages appeared to be the most restricted in terms of interaction. Yes, I had a few clients who wanted me to work online, but the damage had been done. I lost three big opportunities over a 5-month period. This takes a toll especially when you are self-employed.’ (Participant 3, Female, Johannesburg)

Participant 20 was scathing at how some of the restrictions were handled. For instance, this participant was scheduled to work with clients in the food and beverage sector, particularly handing alcoholic products:

‘The food and beverages sector was the worst affected. There appeared to be a so-called expert opinion that called for sporadic changes to this sector during the lockdown phase. So whenever things looked good with trade happening, there was hope. However, this on and off leading to this client of mine calling off my services permanently. The uncertainty was not good for business.’ (Participant 20, Male, Durban)

Another concern was the inevitable possibility of cut-backs. The participating IO Psychologist narrated planning for austerity given the pandemic. This meant the possibility of letting go of support staff especially and even changes concerning rentals of properties where the practice was based. Participant 1 narrated this harrowing ordeal:

‘Austerity was the order of the day. I relied on Road Accident Fund claims, but I considered some serious cut-backs when these dried. These included from cutting hours of support staff to even letting go of one or two of my workers.’ (Participant 1, Female, Johannesburg)

Participant 2 also narrated the same harrowing ordeal, inclusive of personal and logistical cut-backs:

‘As a result of the pandemic, I had seriously considered cutting back. The biggest casualties for me were my staff. It was painful to let staff go but also, because of lack of business, I had to scale back in terms of logistical issues. These included even finding a new premise near Belville that was affordable.’ (Participant 2, Male, Cape Town)

Other participants also expressed indirect effects of the pandemic, noting the challenge of those who work for them, particularly the interns. One participant bemoaned the challenge the pandemic has, especially on assisting the next generation of IO Psychologists:

‘I am not sure if in the next 2 years I will be taking any more interns because of the challenges meted out by the pandemic. Imagine being closed down for 3 months straight, whereas the interns need interaction as part of their experience. So imagine 15 practitioners cutting down on this important aspect of professional development. It can only mean great challenges ahead.’ (Participant 16, Female, Bloemfontein)

A related concern amongst the participating IO Psychologists was the issue of professional development within the profession. It would appear that participants were in praise of the importance of continued professional development, but concerns existed, especially because of the pandemic:

‘So in a year, I take seriously activities concerned with professional development. I attend events, seminars and at least a major conference within the IO Psychology discipline. The past year and as a result of the pandemic, I have cut down on such activities. Yes, I have found a way to make up for these through technology but it’s not the same. So imagine the impact – the pandemic has robbed us of opportunities to develop within the profession. This is sad, especially given the importance of continuous professional development.’ (Participant 8, Female, Johannesburg)

Table 3 presents additional quotes from the participants to the study supporting theme 1 of the study.

**TABLE 3:** Impact of COVID-19: Illustrating quotes.

Impact of COVID-19 on the IO Psychologist as an individual	Impact of COVID-19 on the IO Psychologist as a professional
‘The biggest impact of the pandemic is death. I contracted COVID-19, and to this day, I live with the impact of the pandemic. The journey to recovery has been slow, and I still struggle to breathe at times’. (Participant 25, Male, Johannesburg)	‘I always look forward to some calendar events that help me as a professional. In the year 2020, either they got cancelled, or they were moved online. The implication was limited social contact and cross-sharing of ideas’. (Participant 22, Male, Pretoria)
‘I have not contracted COVID-19 but having to live each day knowing there is a possibility of contracting scares me. Imagine the paranoia and daily routine of cleaning and sanitising. It is necessary, but it also eats one up. It is like a better part of my day is now being spent on being consciously hygienic’. (Participant 13, Female, Pretoria)	‘The pipeline will be affected. From the students wanting to be IO Psychologists. Most of the universities have had to adjust based on the new normal. Then those doing their internships, also affected. Based on what I see, IO Psychology as a discipline may need to be revamped because of the social contact restrictions. We are a profession that needs connection with people. Imagine the impact of cutting this’. (Participant 17, Male, Port Elizabeth)
‘Imagine being in a home of 6, and one of the members gets infected. Life stops. One must now take care of the sick whilst at the same time avoiding being sick. The pandemic year has meant a lot to me. Notably, the need to take care of one’s health. This has become the priority, work comes second after this’. (Participant 11, Female, Johannesburg)	‘In the past few years, I have developed international partners. It is always great to peg with international trends. The travel restrictions have meant we cannot go overseas or even to a country like Namibia. Ultimately, the COVID-19 pandemic imposes not only geographical limits but also professional limits’. (Participant 5, Male, Pretoria)

COVID-19, coronavirus disease 2019.

Based on these presented challenges, the participating IO Psychologists also indicated resultant professional roles that will emerge because of the impact of the COVID-19 pandemic.

### Theme 3: Resultant professional roles because of the impact of the COVID-19 pandemic

The participating IO Psychologists were asked to frame a set of priority professional roles that are important based on the challenges stemming from the COVID-19 pandemic. Four key priority professional roles emerged from the analysed data. These are presented in Table 4 as presented data with illustrating quotes.

**TABLE 4:** Resultant professional roles: Illustrating quotes.

Resultant professional roles	Illustrating quotes
IO Psychologists championing physical and mental health	‘The pandemic period revealed the dual relation between physical and mental health. Being locked down gave some of us time to exercise. I was fit and healthy. Going forward, physical health becomes a continued priority for me’. (Participant 23, Female, Durban) ‘Not working at the normal resulted in some mental strain. One of the things I needed during this difficult period was support concerning my mental well-being. We do not need any pandemic period to make us realise the importance of mental well-being’. (Participant 19, Male, Johannesburg) ‘I think one of the things as a professional body we should be promoting is related to health. The pandemic was a direct threat to our health. Our duty going forward is prioritising this. Not just mental health but also physical health’. (Participant 14, Male, Johannesburg)
IO Psychologists developing technological skills and acumen	‘We need to be more tech-savvy in our work. I am not talking about the basic word-processing skills and developing apps that assist the work we do. This is not necessarily a re-definition of the work but just using new tools to project it more’. (Participant 22, Male, Pretoria) ‘I think a post-COVID-19 world will rely a lot on the use of technology. Given this, from the university system, we need to make sure that the products we are developing not only have this acumen and skills but can still have the same impact whilst using technology’. (Participant 21, Male, Johannesburg) ‘At first, I struggled at the start of the pandemic when everything went online. So with my support team, we empowered ourselves with needed work schedules and including downloading project management software and this helped to monitor what everyone was doing and making it all link into a collective effort. This is something I wish we had explored before’. (Participant 15, Female, Pretoria)
IO Psychologists promoting innovative professional learning and development	‘New themes appear to emerge as a result of the COVID-19 pandemic. One of the things we need to do is to encourage learning within the profession continuously. This includes even imparting skills and knowledge that help us IO Psychologists to be change agents’. (Participant 24, Male, Pretoria) ‘IO Psychologists need to speak to the issues affecting organisations. Emerging themes seeking for organisational responses that are impactful in the midst of difficulty are needed. A precursor is a need for continuous professional development and practitioners that speak to this. This is the challenge going forward’. (Participant 22, Male, Pretoria) ‘I have decided to use the pandemic period for further development. Many service providers like Get Smart are providing such opportunities. These are mostly online, and I know this will come in handy when things normalise. This is something I am encouraging my other colleagues’. (Participant 12, Female, Pretoria)
IO Psychologists developing support networks and structures	‘The pandemic revealed that we are all cut from the same cloth. However, we tend to work in silos. At best, I found reaching out with others during the pandemic helped in terms of a support mechanism’. (Participant 10, Female, Cape Town) ‘Our networks assist us in working together around areas related to the work we do. However, our networks do not extend in dealing with matters of a personal nature or tragedy. I think we need to extend ourselves to be more relational and be there for each other’. (Participant 7, Male, Port Elizabeth) ‘Mental health issues are a reality, especially amongst IO Psychologists. I know of a tragic loss because of such issues. We need to realise that those that help others also need help. So part of the responsibility is for a society of practitioners that help and support each member’. (Participant 6, Male, Cape Town)

COVID-19, coronavirus disease 2019.

## Discussion

The purpose of the study was to ascertain the challenges faced by IO Psychologists as part of their lived experience of the COVID-19 pandemic. In addition to this, the study sought to investigate ensuing roles as a result of the challenges posed by the COVID-19 pandemic. Three main findings emerged: (1) narrated personal challenges as part of the experience of the COVID-19 pandemic, (2) challenges stemming from the COVID-19 pandemic and their impact on the professional sphere and (3) four resultant roles emerged as part of points (1) and (2). This section presents a discussion of these findings with the extant literature.

The presented challenges as illustrated in the findings of this study appear to support views around the drastic changes that happen as a result of the COVID-19 pandemic (World Health Organization, 2020). This study narrowed the focus to a sample group of IO Psychologists in illustrating this change. Notably, the change appears to be a nexus between the personal and professional identity of the IO Psychologists. This link supports the theorising of Raju and Van Niekerk (2020) as to the idea of the compounding effect of the pandemic. In essence, through this change, the findings of the research attest to the acknowledged impact of the pandemic on how people work and how they live their lives (Bapuji et al., 2020).

The two presented challenges of the COVID-19 pandemic to the personal and professional identity potentially can pose a threat to the health and well-being of the IO Psychologists (Beckman et al., 2021). This has the potential to create both psychological and physical strain on the individual (Bakker & Demerouti, 2007). Addressing these personal and professional challenges stemming from the COVID-19 pandemic creates a platform to assist practitioners (Kelly, 2020). Whilst going through the identified challenges, focus should be on ensuing roles as a mechanism to assist IO Psychologists during this period of difficulty. The resultant roles appear to be framed on the consideration of the interaction between individual and social experiences (Carlson et al., 2017). In essence, findings appear to support some presented theoretical positions. For instance, the importance of human capabilities such as IO Psychologists as framed in the human capital theory (Bag & Gupta, 2019). There is a need to understand the challenges faced by such human capabilities and the means of coping (Hofmann & Rüsch, 2017). The findings of the study illustrate the importance of IO Psychologists as crucial human capabilities and the challenges and resultant roles of coping.

The study prioritises four roles as a response to the COVID-19 pandemic. These roles include: (1) the prioritisation of personal physical and mental health, (2) more technological skills and acumen needed to adjust to the challenges posed by the pandemic, (3) promotion of continued professional learning and (4) the necessity for support networks amongst practitioners. These roles should be seen as resources of coping with personal and professional situations. In essence, attesting support to the job demands-resources model (Bakker & Demerouti, 2007). The resultant roles can be seen to support the idea of Ryff (2013) of resources that can assist an individual to promote their well-being.

The study makes some important contributions. Firstly, through the resultant four roles of IO Psychologists because of the challenge of the pandemic, the answers call for research seeking an understanding of the new normal of working given the challenges of the pandemic (Handfield et al., 2020; Trautrims et al., 2020). Secondly, the findings of the study potentially extend understanding around emerging work on the impact of the COVID-19 pandemic not only at a societal level but also with a disciplinary angle (De Klerk et al., 2020; Magezi & Manzanga, 2020). This has the potential to create a body of knowledge from which interventions can be made (Pillay & Barnes, 2020) especially within an important discipline such as Industrial Psychology (De jager-van Straaten et al., 2016). Given that the work of IO Psychologists is community centred, the presented findings assist in creating points of helping practitioners with their important role in the community. Thirdly, the presented professional roles (considering also the presented challenges) have the potential to help IO Psychologists shape the future themes of their profession in general and also to promote their general well-being. For instance, the promotion of technological acumen in the work of IO Psychologists, as found in this study, becomes a skill being recognised as important both in pre- and post-COVID-19 world. Technology such as telecommuting, virtual teams and cloud computing can then enhance the work of IO Psychologists (Li, 2016).

## Implications

Based on the findings of the study, some implications can be drawn. Firstly, there is a need to provide support services to IO Psychologists and their families in response to the pandemic’s challenges. Despite IO Psychologists often being the people who provide support services, they also need a platform through which these support services can exist. Secondly, there is a need to prioritise the continuous professional development of IO Psychologists, especially during the pandemic. This development can be useful for the benefit of the IO Psychologists and for the profession. Thirdly, professional associations such as the Society for Industrial Organisational Psychology South Africa can take a further active step in providing a platform of expression for IO Psychologists. This can be to assist in providing support services and structures that help professionals. Responsibility should also be given to the IO Psychologists to develop skills and acumen needed to survive, especially in a post-COVID-19 world. These skills and acumen can be technological in nature and become important, especially given the global shift towards more online-based working. Implications can also be drawn based on how the next generation of IO Psychologists can and should be trained. Emphasis is on a balance between skills that assist in advancing the individual and those of the profession. This calls for a re-think of the IO Psychology curricular being taught, especially in universities. A need here is for a curricular that empowers the IO Psychologist in training to be ready for the ever-changing work context and for the world in general.

## Limitations of the study

Some limitations can be drawn from the study. Firstly, caution should be exercised when interpreting the study’s findings, given the apparent challenges concerning sampling. The research relied on a convenience sample and does not represent all the registered IO Psychologists in South Africa. Secondly, all of the interviews were conducted online. Although this created an opportunity to actually conduct the interviews, it was a process that had its own difficulty. In some cases, connectivity challenges emerged as a problem as most participating IO Psychologists worked from home. This created a challenge both around connectivity and the need to accommodate participants as they juggled between work and home demands. In some cases, some of the participating IO Psychologists requested the researcher to purchase data bundles to allow the interviews to take place. A final challenge concerned issues around the period during which the interviews were conducted. The data collection happened 2 months after the first lockdown announced by President Cyril Ramaphosa in March 2020. From December 2020, South Africa was believed to be going through a second variant of the COVID-19 pandemic. Furthermore, from January 2021, there was talk of a vaccine roll-out. These are important events happening. The data collection conducted during this study did not factor in these events. In essence, the framing of the impact and resultant roles to emerge were mostly confined to the pandemic’s early experiences. Furthermore, there is also the challenge of retrospective bias, especially when asking participants to recall their experiences.

## Future research

Suggestions for future research can be made. Firstly, this study explored general challenges faced by IO Psychologists; there is thus a need to narrow focus on specific challenges and explore these further. For instance, an in-depth study could be warranted to understand aspects of work-home life balance amongst IO Psychologists especially during the pandemic period. Another interesting area of future research could be to explore the support mechanisms IO Psychologists rely on not necessarily as part of professional development but as individuals facing the pandemic challenges. Secondly, there is a need for more studies that could also incorporate other stakeholders who may work under the IO Psychologists’ supervision. These could include interns and gauging their experiences related to their development in becoming professionals within the IO Psychology discipline. This can also provide useful information that assists in improving the practice in general. Future research can categorise the experience of IO Psychologists, considering the different levels and areas of expertise. This study provided a more general outlook, yet this narrowed focus can also help to understand the experience better within the same cohorts. Finally, new scales are being developed that are specific to COVID-19 concerns and perceptions. It would be interesting to conduct quantitative-based surveys that explore such experiences. This would further help improve understanding of the impact of the COVID-19 pandemic within the confines of the IO Psychology discipline.

## Conclusion

The study thus makes an important contribution to an important topic that deserves further interrogation. An opportunity here through understanding the challenges presented in this study could be proposing interventions that assist IO Psychologists in their personal and work roles. Through the resultant professional roles, the study reveals priority issues as developmental in the work performed by IO Psychologists.
